# Does First-Use Syndrome Happen During Apheresis? A Case Report

**DOI:** 10.7759/cureus.39126

**Published:** 2023-05-17

**Authors:** Ashraful Hoque, Sushanta K Basak, ABM Al Mamun

**Affiliations:** 1 Infectious Disease, Sheikh Hasina National Institute of Burn & Plastic Surgery, Dhaka, BGD; 2 Transfusion Medicine, Sheikh Hasina National Institute of Burn & Plastic Surgery, Dhaka, BGD; 3 Medicine, Dhaka Medical College Hospital, Dhaka, BGD

**Keywords:** skin blister, citrate toxicity, ethylene oxide, first use syndrome, platelet apheresis

## Abstract

Steam, dry heat, radiation, ethylene oxide gas, evaporated hydrogen peroxide, and many other sterilization methods are used to sanitize medical equipment (e.g., chlorine dioxide gas, nitrogen dioxide, and vaporized peracetic acid). The benefits of ethylene oxide (EO) are its great processing capabilities, high ionic conductivity, high flexibility, low cost, and exceptional adhesive qualities. Patients on hemodialysis, those undergoing extracorporeal photopheresis, and plasmapheresis donors have all reported allergic reactions to EO. Differentiating between IgE-mediated anaphylaxis and anaphylactoid reactions is often impossible in practice due to the wide range of clinical symptoms. The infrequency of EO reactions coupled with healthcare personnel's lack of familiarity with this clinical phenomenon may result in their underdiagnosis. We describe the case of a platelet donor who developed an allergy, while donating at a transfusion facility, due to an ethylene oxide-sterilized apheretic kit. We aim to draw attention to the fact that care should be given while handling cases of this nature as they can become life-threatening.

## Introduction

Ethylene oxide (EO) is a hazardous gaseous substance used to sterilize a diverse range of medical equipment that cannot withstand conventional autoclave sterilization methods. Heat-labile plastics, such as intravenous tubing and commercial apheresis kits used for apheresis, photopheresis, and blood component collection, are frequently sterilized using EO. Poothullil et al. first described the case of a chronic hemodialysis patient who had typical systemic allergic reactions to EO via EO-specific IgE [1]. Dialysis patients, plateletpheresis, plasmapheresis, photopheresis, cardiac bypass, EO-sterilised cardiac stents, and blood transfusion through EO-sterilised leukofiltration filters have all been linked to allergic reactions to EO [2,3]. Due to ignorance and a lack of awareness, such allergic reactions may go unrecognized because they are quite uncommon. We describe a case of the first-use syndrome, an allergic reaction to EO, that happened during platelet collection in an apheretic donor that is much more likely to happen after starting hemodialysis.

## Case presentation

On October 1, 2022, a male donor of apheretic platelets, aged 22, presented at the Sheikh Hasina National Institute of Burn and Plastic Surgery. The individual in question did not consume any substances and did not possess any medical history pertaining to conditions such as asthma or drug sensitivity. A complete blood count (CBC) conducted before the procedure was verified to be within the normal range. As per instructions, a dosage of 1 gram of calcium was orally administered. The apheresis procedure was conducted utilizing a Haemonetics MCS+9000 instrument (Haemonetic Corporation, Braintree, Massachusetts) that was primed with an ACD-A solution produced by the same manufacturer. Following the completion of a single cycle, the donor exhibited symptoms of dyspnea, diaphoresis, and loss of consciousness. Vital signs were consistent with anaphylaxis: pressure was 60/40 mm of hg, pulse was 120 b/min, and respiratory rate was 36 breaths per minute. The administration of crystalloid saline was initiated for the patient's care. After administering 500 ml of saline, blood pressure and pulse were measurable. Patients became conscious again. No steroid was given. The procedure was ceased as a result of unforeseen risks. A repeated CBC was conducted which did not reveal any statistically significant alterations, such as an increase in eosinophil count. 

The individual exhibited the formation of blisters in the vicinity of their oral aperture, nasal cavity, and superior eyelid region. There were no lingering symptoms following recuperation. Prophylactic measures were taken by administering a topical antibiotic. The laboratory tests for C-reactive protein (CRP) and CBC were performed as part of the standard procedure. After a period of seven days, the blisters had subsided and no residual symptoms were observed.

**Figure 1:**
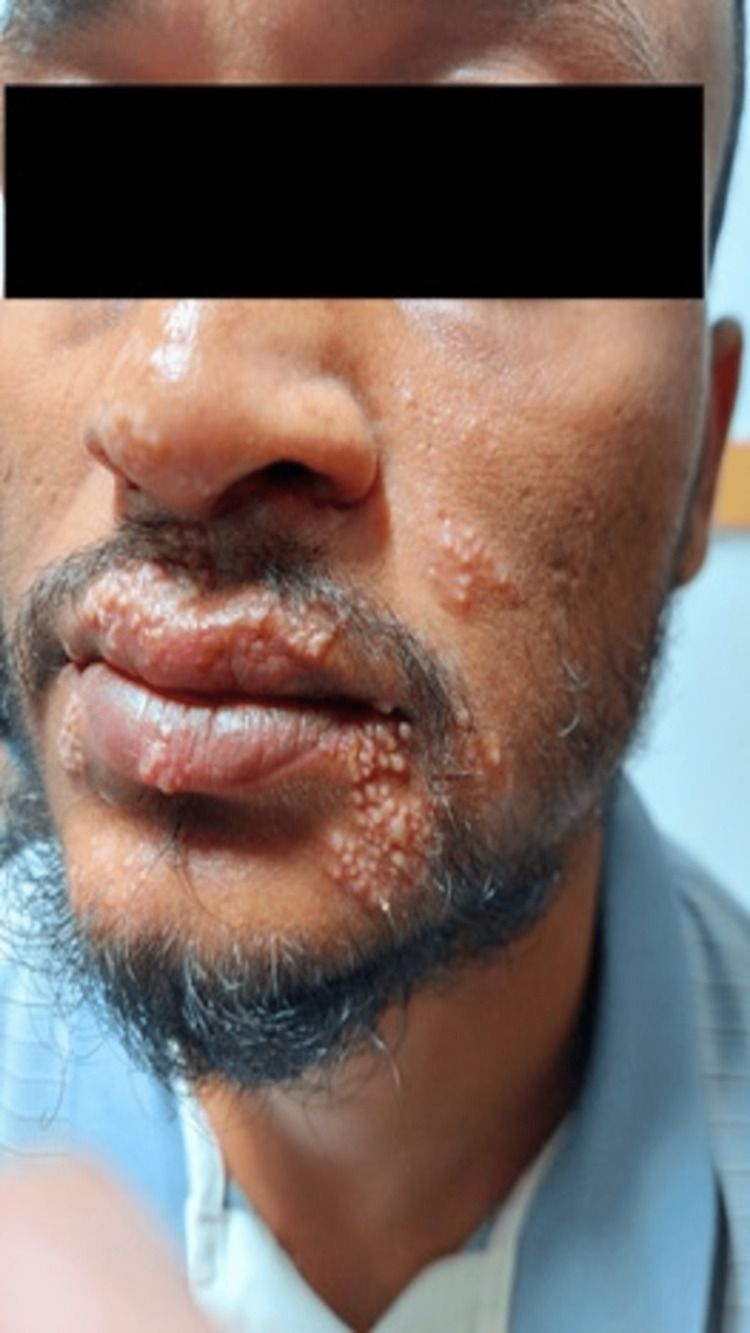
Skin blisters.

The toxicity status of the patient after the development of blisters around the oral aperture, nasal cavity, and superior eyelid region is presented in Figure 1.

Investigations

A complete blood count, serum electrolytes, serum calcium, serum IgE, serum IgA, and plasma basophil were sent right away. Eosinophilia was discovered in the blood count. Serum electrolytes and calcium levels were normal. Serum IgE levels were 800UI/ml, and plasma basophil levels were 1.5ng/ml. Serum IgA levels were normal. 

Differential diagnosis

A normal serum calcium count ruled out citrate toxicity.

Impact and follow-up

The patient was admitted to a local hospital and carefully monitored. Only intravenous fluid was kept in, no oxygen support was needed. After 12 hours, he developed skin lesions that looked like pustules on his lips, nasal septum, lower eyelids, ear lobules, and oral cavity. Neutrophil and white blood cell counts were high, prompting the use of an oral antibiotic. Decongestants were put in the noses of the patients. The dentist recommended using mouthwash to help with oral hygiene. After two days, the lesions had faded and he was feeling better. The patient was referred to the Department of Allergies. Even though the radioallergosorbent test (RAST) was negative, it was determined that the reactions might be attributable to a type I hypersensitivity reaction to EO based on provocation and elimination testing.

## Discussion

EO is a volatile and toxic ether that is commonly utilized for the sterilization of various medical devices and plastics at low temperatures [4,5]. EO easily transforms into polyethylene glycols, ethylene chlorohydrin, and water when combined with polyvinyl chloride (PVC). The fact that EO reacts with and modifies amide (-NH2), sulfhydryl (-SH), and carboxyl groups (-COOH) by adding a terminal ethyl alcohol moiety is more significant. The outcome entails the deactivation of organic matter and the creation of haptens. Consequently, exposure to EO has the potential to elicit an immunological reaction which can manifest as contact dermatitis in the case of oxygen masks and gauze, as well as type 1 hypersensitivity allergic reactions. The list of possible side effects from exposure continues with dizziness, headaches, vomiting, convulsions, blistering, vomiting, and coughing. Even when combined with human albumin, EO can cause an allergic reaction in some people. Sensitization occurs when microparticles from an apheretic kit are left behind. Effector cells like mast cells and basophils react to IgE receptors, setting to anaphylaxis. During the sensitization stage of type I hypersensitivity, allergens (or antigens) are delivered to T-cells by antigen-presenting cells (APCs). IgE antibodies attach to Fc receptors on mast cells and basophils after being stimulated to be produced by T cells. The bound IgE antibodies on mast cells and basophils are then cross-linked by the free antigen. Consequently, the cells degranulate and produce mediators including histamine and proteolytic enzymes (i.e., prostaglandin, cytokines, leukotrienes, platelet-activating factors, macrophage inflammatory proteins, tryptase, etc.). Consequences include a rise in mucus discharges, bronchospasm, stomach cramps, rhinitis, and possibly hypovolemia or hypoxia due to peripheral vasodilation and smooth muscle contraction. Fluid shifts into the interstitial space which can lead to pulmonary edema or systemic edema. Pruritus and local response to asthma, or anaphylaxis can cause a systemic reaction in certain people [6].

The initial documentation of EO reactions in relation to extracorporeal procedures dates back to 1971 when three pediatric cardiac surgery patients experienced unforeseen fatalities [5,7]. Subsequently, chronic dialysis patients in the 1980s experienced a sequence of type 1 hypersensitivity reactions which encompassed anaphylactic shock. It is noteworthy that EO reactions were observed primarily during the initial utilization of EtO-sterilized, reusable hollow-fibre dialysis filters. Within the identical time period, a limited number of instances were documented regarding adverse reactions to EO in relation to automated donor apheresis. Six healthy allogeneic repeat plateletpheresis donors were used in the first and largest report which was obtained using the Baxter CS-3000 (Fenwall Laboratories, Deerfield, Illinois). A similar case was then reported a few months later [8,9]. 

EtO reactions are generally uncommon. The historical EtO reaction rate in dialysis was estimated to range between 0.02% and 0.008% per procedure and 2% in patients undergoing chronic dialysis. According to a previous investigation on plasmapheresis donors, the likelihood of experiencing an EO reaction during apheresis was estimated to be 0.002% per procedure [3]. Laura Cooling at the Department of Pathology, Transfusion Medicine, University of Michigan Hospitals, USA, calculated the risk of an EO reaction at 0.08% per hematopoietic progenitor cell collection (HPCC) procedure and 0.18% per donor based on a 10-year evaluation of HPCC at that facility [10]. This is the first case in our facility and most probably the first case report from Bangladesh.

## Conclusions

The infrequency of EO reactions may result in their oversight, particularly among less experienced personnel who possess limited familiarity with this phenomenon. In contrast to the majority of EO reactions documented in the literature, the patient under consideration did not exhibit a medical history of asthma, atopy, prior apheresis, or dialysis. Our findings indicate a lack of correlation between elevated eosinophil counts and symptoms associated with eosinophilic esophagitis. It is advisable to take into account the possibility of EO hypersensitivity in patients who exhibit symptoms resembling an allergic reaction during the initial 15 to 30 minutes of apheresis procedures. In the medical community, apheretic platelets have gained popularity since they require fewer donors than random preparation and provide patients with better results. For the safety of the donors, management of anaphylaxis in accordance with the standard operating procedure should be established at every transfusion center. 
